# Optimizing the Composition of Silicone Enamel to Ensure Maximum Aggregative Stability of Its Suspensions Using Surfactant Obtained from Oil Refining Waste

**DOI:** 10.3390/polym14183819

**Published:** 2022-09-13

**Authors:** Vitaliy Tyukanko, Alexandr Demyanenko, Antonina Dyuryagina, Kirill Ostrovnoy, Gulsim Aubakirova

**Affiliations:** Department of Chemistry and Chemical Technology, M. Kozybayev North Kazakhstan University, Petropavlovsk 150000, Kazakhstan

**Keywords:** optimization of composition content, aggregative stability, Taguchi method, mathematical modeling, surfactants, silicones, polyorganosiloxanes, organic coatings

## Abstract

The aim of this study was to optimize the composition of enamel consisting of aluminum pigment and polyphenylsiloxane polymer, in order to achieve the maximum aggregative stability of suspensions. Sedimentation rate (SR) was used as a criterion for assessing the aggregative stability of the suspensions. An original product, AS-1, and industrial additives PEPA and Telaz, were tested as surfactants. AS-1 was obtained from oil refining waste at M. Kozybayev North Kazakhstan University. All the studied surfactants improved the stability of the suspensions. The AS-1 additive significantly improved the stability of the suspensions, but exhibited a lower stabilizing ability by 10–20% than PEPA. The maximum overall stability of the suspensions was recorded at a PEPA level of 0.25–0.375 g/dm^3^ in the enamel. The Taguchi method was used to optimize the composition of the enamel, using AS-1 as the surfactant. It is recommended to use AS-1 in silicone enamels. Optimum compositions can reduce the petrol absorption of coatings by 1.5 times, their roughness by 2.5 times and increase their gloss.

## 1. Introduction

Currently, two-component silicone enamels with excellent anticorrosive properties of coatings available at a relatively reasonable cost are widely used in industry [1]. These enamels consist of silicone varnish and aluminum pigments that are mixed immediately before use. This mixing creates difficulties in assessing the stability of the obtained enamels, since over time they become stratified into a pigment and a layer of varnish. When applied, the pigment particles agglutinate, and the protective properties of the coatings deteriorate significantly. The size of the pigment particles is a very important characteristic of the silicone coating, which determines its anticorrosive (permeability as well as insulating and adhesive abilities), decorative (color, gloss) and structural/mechanical (opacity, porosity, hardness, tensile strength and impact) properties in coatings. Thus, the introduction of nanoparticles of various materials (metals, titanium dioxide) significantly increases the anticorrosive properties of silicone coatings [2] and polymer composites [3,4].

In order to reduce the processes of aggregation of pigment particles that occur when using two-component enamels, various surfactants are used. At the same time, it is interesting to note the positive effect of various surfactants on improving the aggregative stability of “pigment-silicone-solvent” suspensions. A significant amount of research has been devoted to investigate the effect of surfactants on the aggregative stability of suspensions in different environments [5,6,7,8,9,10]. The effectiveness of surfactants on the stability of suspensions obtained during dispersion (assessing their aggregative stability) is determined by making rational choices based on the literature data.

For years, polyester and alkyds have been well known to be surfactants in silicone enamels [11,12]. The introduction of a nitrogen-containing surfactant significantly increased the adhesion of the silicone matrix (oligomethylsiloxane) to the filler, thus improving the characteristics of the composite [13]. The positive effects of the resulting formation of an antifouling coating from the nitrogen-containing compound—2-(2-benzimidazolil) ethantiol in silicone enamel were shown in [14]. The introduction of benzotriazole and nanosilicon (as a filler) into a polydimethylsiloxane composite significantly improves the anticorrosive properties of coatings [15]. The introduction of an adhesion enhancer additive into a silicone composite significantly improves its operational characteristics [16]. In the literature, we found information about the development of silicone composites with the following nitrogen-containing additives: aniline oligomer [17], 3-aminopropyltriethoxysilane [18], erucamide [19], melamine [20], polyamidoamine [21], amines [22] and polyimides [23]. In addition, the introduction of surfactants into paints not only improves the dispersion of the pigment [24,25], but also improves the wetting of pigments as well as the steel surfaces of painted parts, with their solutions [26,27,28,29].

For the effective selection and use of additives in specific silicone paint and varnish materials (primers, enamels, etc.), it is necessary to conduct in-depth research in the field of colloidal and physical chemistry. However, in practice, we often observe only compliance with the recommendations of additive manufacturers with “approximate additive costs”. Currently, a large number of reference books and catalogs of manufacturers of various additives are known [30], which are used by technologists who create paint formulations in industry. However, the information in these sources about the use and optimal content of additives in coatings is purely empirical. Currently, there is no generally accepted “unified scientific theory of additive selection” for various coatings, depending on the polarity of the polymer and solvent, their solubility parameters, the surface properties of pigments and fillers, etc. Although scientific attempts are being made to systematize this issue, in particular, the research of [31] provided a complete analysis of surfactants in coatings used throughout the entire period of human development, from ancient times to the present day. The authors focused on the physico-chemical bases pertaining to the use of surfactants in composite materials, including in coatings. The influences of the types and concentrations of surfactants on the change in interphase energy and the stability of dispersed systems were shown. It has been proven that using surfactants in enamels and paints improves the aggregative and sedimentation stabilities of suspensions/dispersions. The study [32] showed that the introduction of surfactants into the paintwork causes an increase in the number of adhesive “organic coating—pigment” and “organic coating—steel surface, painted parts” contacts; as a result, the protective properties of the paintwork increase significantly. This article has been a part of our research at North Kazakhstan University since 2009 on the development of a “unified scientific theory of additive selection” for various coatings, such as silicone, pentaphthalic, oil, bitumen, perchlorvinyl, water-acrylic, urethane-alkyd, etc.

In summary, the use of amino surfactants to enhance the overall stability of suspensions in silicone compositions looks very promising. Due to the growing global economic crisis (the COVID-2019 pandemic and large inflation of reserve currencies such as the US dollar and the Euro), the demand for cheap additives obtained from petrochemical waste is increasing significantly. In this study, the AS-1 product obtained from the petrochemical refinery waste at M. Kozybayev North Kazakhstan University was used [33]. In order to evaluate the overall stability of the suspensions in the presence of the original product AS-1 compared to industrial additives, we used additional additives: “Telaz” and “PEPA”. Previously, we proved the effectiveness of these surfactants (PEPA, Telaz, AS-1) as dispersant additives in silicone enamel [34,35]. However, due to the two-component nature of this enamel (it is obtained immediately before painting by mixing varnish and dry pigment), the aggregative stability of pigment suspensions during their storage was not taken into account. Consideration of this factor is necessary to make the correct choice of the type and content of surfactants in the studied enamel. Sometimes, there is a good dispersal ability of surfactants accompanied simultaneously by low stabilizing activities against the pigment suspension. As a result, during enamel storage, pigment suspensions settle quickly, particles agglutinate and organic coatings with a significant number of large pores form.

In order to avoid these phenomena from occurring, we assessed the overall stability of suspensions in this article by measuring the sedimentation rates of the suspensions (SR) of the enamel under investigation. SR allows us to take into account the influence of the content of not only surfactants, but also of solvents, on the aggregate stability of suspensions, and to quantify these changes in order to mathematically optimize the composition of the enamel.

## 2. Materials and Methods

### 2.1. Materials

**Figure 1:**
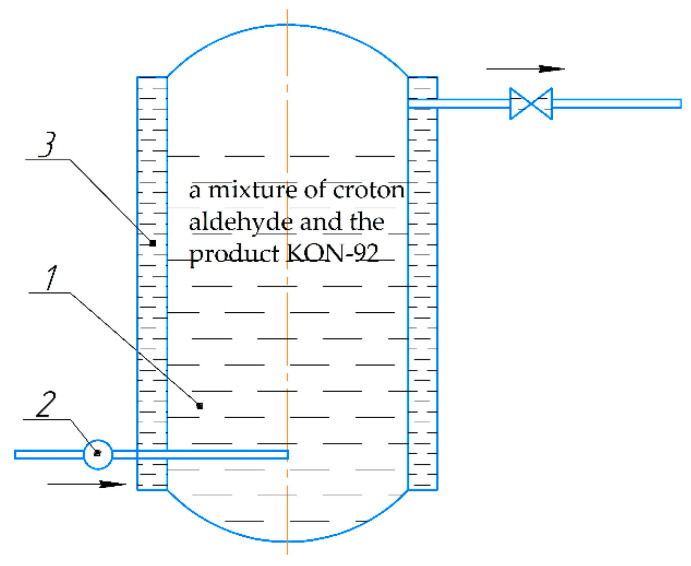
Technological scheme for obtaining the AS-1 additive (1—reactor; 2—gas reducer; 3—water/cooling jacket).

In this study, polyphenylsiloxane KO-85 based on GOST 11066-74 (hereafter referred to as PPhS) was used as the primary polymer (film-forming agent). Toluene was used for the dissolution of PPhS. This brand of toluene was characterized as follows: density—0.866 g/cm^3^ (at 20 °C); impurity content—no more than 0.2% (the rest is toluene). In silicone enamel, aluminum powder of the PAP2 brand was used as a pigment. This pigment contains no more than 3% impurities (of which no more than 2.5% constitutes fat). The following nitrogen additives were used in the study:

(1) AS-1 additive. It was obtained (synthesized), according to the technological scheme shown in Figure 1, from two main components: cubic residues of petrochemicals KON-92 and croton aldehyde.

The first component is the cubic residues of the production of butyl alcohols and 2-ethylhexanol, under the trademark KON-92 (TU 38.302-75-03-92), which is a mixture of aldehydes (acetic—5.6%, oil—4.4%, croton—1.1%, 2-ethylbutenal—12.8%, hexanal—2.4%) and alcohols (2-ethylhexenol—2.3%, isohexanol—1.6%, 2-ethylhexanol—4.0%, butanol—the rest). The second component is industrial croton aldehyde (C_4_H_6_O), with a base substance content of 98.3%. In the reactor (item 1) made of AISI 316 steel, with a volume of 0.5 dm^3^ (maximum filling factor—0.7), KOH-92 was poured in the amounts of 300 g and 75 g of croton aldehyde. Then, industrial ammonia (NH3) was fed into the reactor according to GOST 3760-79 under a fixed partial pressure of 0.025 MPa (due to the gearbox pos.2) from a gas cylinder. The temperature in the reactor (25 ± 2 °C) was maintained using water circulation through a water jacket and heat exchangers pos.3. The synthesis time was 55 min. As a result, the additive AS-1 was obtained, in the amount of 375 g. A mixture of primary and secondary amines was obtained (molecular weight—250 a.m.u.; amine number (mg HCl/g)—30) [33].

(2) Additive “Telaz D” (molecular weight—2121 a.m.u.; amine number (mg HCI/g)—32). “Telaz” is a condensation product of vegetable oils with diamines.

(3) “PEPA” additive is a mixture of high molecular weight amines (molecular weight—4950 a.m.u.; amine number (mg HCI/g)—31).

**Figure 2:**
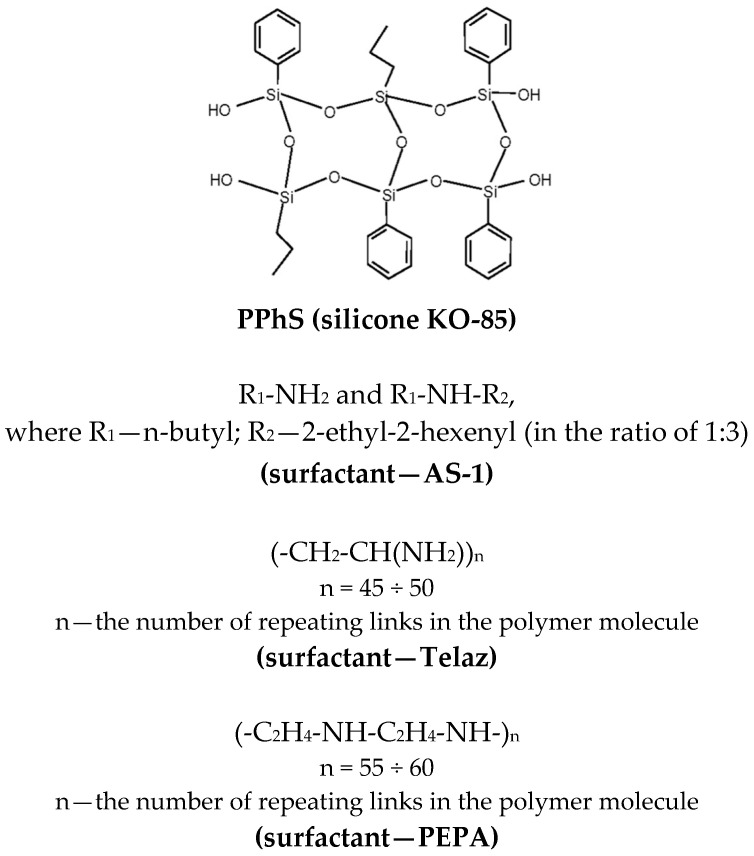
Chemical structures of the used compounds.

The structures of the base polymer and nitrogen-containing surfactants are shown in Figure 2.

### 2.2. Measurement of Aggregative Stability of Suspensions “Aluminum Pigment-PPhS-Toluene”

The aggregative stabilities of the aluminum pigment and toluene suspensions were measured by calculating the sedimentation rate in meters/second (SR). The aggregative stabilities of the “aluminum pigment-PPhS-toluene suspensions” were measured by calculating the sedimentation rate in 10^−2^ m/3600 s (10^−2^ m/h). All measurements were taken at least five times, and the average values are reported. The measurements were carried out at a temperature of 20 ± 2 °C.

### 2.3. Experimental Design

The Taguchi method was used to identify the influence of each optimization factor (surfactant content and solvent content) on the aggregative stabilities of suspensions—sedimentation rate (hereinafter SR). The Taguchi method makes it possible to identify the optimal combination of optimization parameters in order to obtain composites with the necessary parameters [36,37]. In our case, it was necessary to obtain a two-component silicone enamel with maximum aggregative stability of the “Pigment-Surfactant-Toluene-PPhS” suspensions.

In the course of the study, compositions were used in which there were variations in the following:

1. The content of surfactant AS-1 (hereinafter CS)—from 0 to 0.75 g/dm^3^;
2. The solvent content (hereinafter referred to as SC)—from 10 to 30% (by weight of the composition).

Table 1 shows the factors under consideration and their corresponding levels [37].

#### Planning an Experiment Using the Taguchi Method

In this study, the L9 matrix was used, and the signal-to-noise ratio (S/N) was used as a characteristic of the selection quality. In the Taguchi method, three different functions are used to find the (S/N): “the-smaller-the-better (SB)”, “the-nominal-the-best (NB)” or “the-larger-the-better (LB)”. In order to minimize SR, we used the SB function (Equation (1)) [34], shown as follows:

(1) $$S/N=-10\log_{10}\left(\frac{1}{n}\sum_{i=1}^{n}y_{i}^{2}\right)$$

where *n* is the number of test runs, and *y_i_* are the measured values of SR.

The S/N ratios for SR were calculated according to Equation (1), as shown in Table 2.

### 2.4. Methodology for Checking the Quality of Coatings

The effects of the surfactants on the quality of the silicone coatings were assessed by their effects on the decorative properties (gloss, film roughness) and protective properties (petrol absorption and resistance to seawater) of the coatings.

The gloss of the coatings was determined in accordance with the ISO 2813:1994 standard on the FB2 gloss meter on steel plates 150 × 70 × 0.2 cm (length × width × thickness). The coatings were applied to the plates by pouring, and were maintained for 8 h at a temperature of (350 ± 5) °C. All of the samples were examined at least 5 times. The reported results show the mean values.

The roughnesses of the films were determined using a double MIS-11 microscope (according to a standardized technique) on steel plates 150 × 70 × 0.2 cm (length × width × thickness). The coatings were applied to the plates by pouring, and were maintained for 8 h at a temperature of (350 ± 5) °C. All of the samples were examined at least 5 times. The reported results show the mean values.

The petrol absorption and moisture absorption of the coatings were determined using the gravimetric method (weighing the plates with the coatings before and after exposure in petrol/distilled water for 20 days) on steel plates that were each 65 × 25 × 0.1 cm (length × width × thickness). The coatings were applied to the plates by pouring, and were stored for 8 h at a temperature of (350 ± 5) °C. All of the samples were examined at least 5 times. The reported results show the average values.

The effects of the additives on the stabilities of the coatings to water of the Caspian Sea were assessed by external inspection. The tests were carried out on steel plates that were 65 × 25 × 0.1 cm (length × width × thickness) each, at a temperature of (18 ± 2) °C for 168 h. The coatings were applied to the plates by pouring, and were stored for 8 h at a temperature of (350 ± 5) °C.

### 2.5. Adsorption Measurements

The degree of PPhS and surfactants adsorption on the surface of the aluminum pigment was calculated from the difference in concentrations before and after the adsorption measurements, using a calibration curve. The adsorption was determined by photocolorimetry methods (on the KK-3-01 device, by measuring the transmittance (T, %) wavelengths at 400, 440 and 490 nm), and by measuring the surface tension (σ, J/m^2^). The first stage of the measurements was the preparation of PPhS/aluminum pigment suspensions in the same systems, additionally enriched with surfactants. A suspension of aluminum pigment (0.4 g) was added to 25 cm^3^ of a solution containing PPhS, and in some surfactant systems (concentration range from 0 to 2 g/dm^3^). The suspensions were mixed for 30 min at a temperature of (20 ± 2) °C in a sealed reactor (volume (0.2 ± 0.01) dm^3^, filling factor—0.60) that was equipped with a mixing device (impeller agitator, speed—300 min^−1^) in order to achieve adsorption–desorption equilibrium.

## 3. Results

### 3.1. Effects of Surfactants on the Aggregative Stability of Suspensions “Aluminum Pigment-PPhS-Toluene”

**Figure 3:**
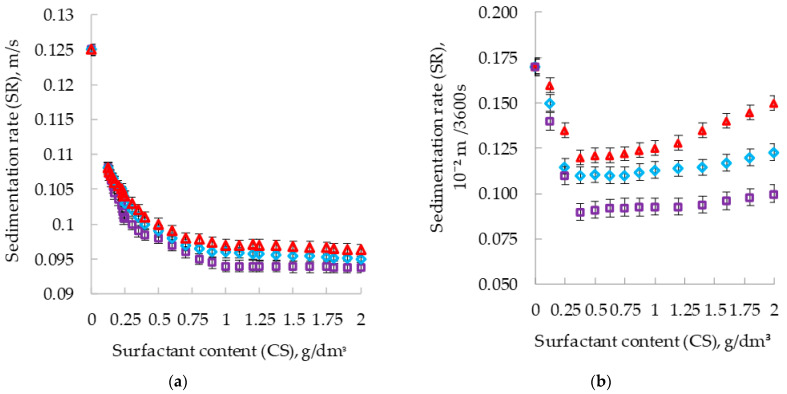

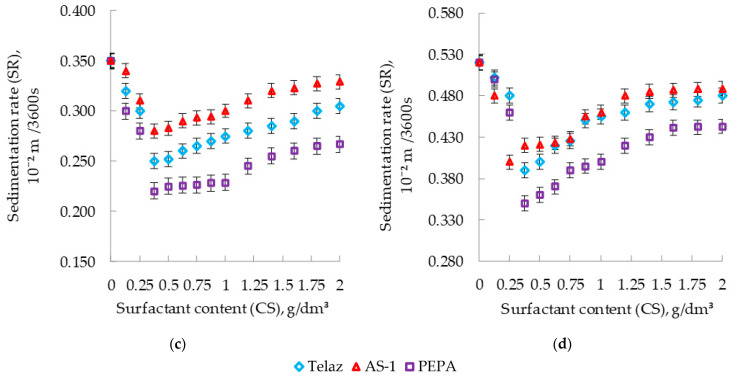
Effects of the type and content of surfactants on the sedimentation rates of aluminum suspensions (**a**) in pure solvent (SC 100%); (**b**) with a solvent content of 10% (SC 10%); (**c**) with a solvent content of 20% (SC 20%); (**d**) with a solvent content of 30% (SC 30%).

The effects of the surfactants on the sedimentation rates of suspensions in pure toluene are shown in Figure 3a, and in the presence of PPhS in Figure 3b–d.

**Figure 4:**
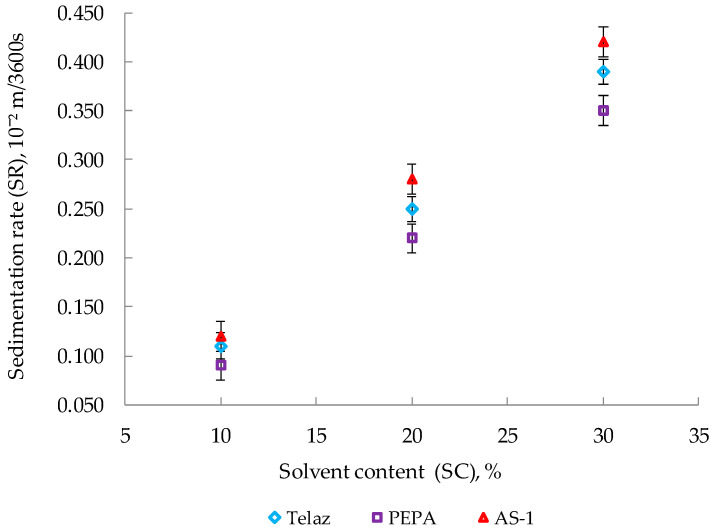
Effects of the type of surfactants and the solvent content on the deposition rates of aluminum suspensions (with a surfactant content of 0.375 g/dm^3^).

The effects of the solvent content on the sedimentation rates of the silicone enamel suspension are shown in Figure 4.

### 3.2. Optimization of Aggregative Stability of Suspensions by Taguchi Method

**Figure 5:**
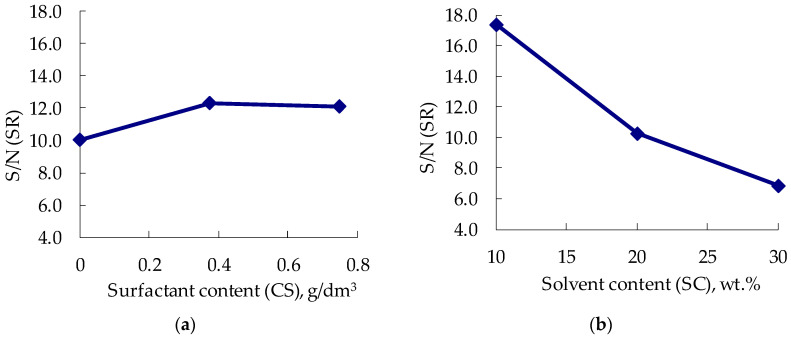
Main effect plot for signal-to-noise ratio: content of the surfactant (CS) for SR (**a**), solvent content (SC) for SR (**b**).

The average values of the signal-to-noise ratio for the SR for each parameter level for the AS-1 are shown in Table 3, and these values are illustrated by the graphs of the main effects shown in Figure 5a,b.

The highest point on the graph indicates the level to be selected for the optimum parameter. The rank and delta values indicate the most significant parameter. The delta values for each parameter show the difference between the highest and lowest values of the SR.

From Table 3, the solvent content (SC) was found to be the most significant parameter affecting the SR, followed by surfactant content (CS). Figure 5a,b show that the addition of 0.375 g/dm^3^ of AS-1 to the composition resulted in the highest aggregate stability of the suspensions/pigment dispersion for SC 10%. In order to evaluate the contribution of each factor to the aggregative stability of the suspensions, an analysis of variance (ANOVA) was conducted, and the results of the calculations are presented in Table 4.

ANOVA analysis for the S/N ratios in Table 4 showed that the solvent content (SC) (contribution of 94.83%) affected the aggregate stability (SR) more than the surfactant content (CS) (contribution of 5.17%).

In order to derive a multifactorial statistical mathematical model of the effects of surfactants and solvent content on the SR, the following equation proposed by M. M. Protodiakonov (2) was used:

(2) $$Y_{0}=\frac{\prod_{i=1}^{p}Y_{i}}{{\bar{y}}^{p-1}}$$

where $Y_{0}$ is the generalized equation; $Y_{i}$ is the particular function; $\prod_{i=1}^{p}Y_{i}$ is the product of all particular functions; *p* is the number of particular functions equal to the number of input parameters; and $\bar{y}$ is the average value of the output parameter for all *n* experiments (the general average).

The reliability of the obtained mathematical model was determined by calculating the coefficient of nonlinear multiple correlation (3) [31]:

(3) $$R=\sqrt{1-\frac{\left(n-1\right)\cdot \sum_{i=1}^{n}{\left(y_{i}-{\hat{y}}_{i}\right)}^{2}}{\left(n-p-1\right)\cdot \sum_{i=1}^{n}{\left(y_{i}-\bar{y}\right)}^{2}}}$$

where *n* is number of experiments; *p* is number of input (independent) parameters; *i* is serial number of the experiment; $y_{i}$ is the actual value of the output parameter in the *i* experiment; ${\hat{y}}_{i}$ is the calculated value of the output parameter, calculated using a multi-factor mathematical model, for the conditions (values of input parameters) of the *i* experiment; $\bar{y}$ is the average value of the output parameter for all *n* experiments (the general average).

After approximating the dependencies using Equation (2) and Microsoft Excel, the following equation for SR (4) was obtained:

(4) $$\mathrm{SR}=\frac{\left(a\cdot {\mathrm{CS}}^{2}+b\cdot \mathrm{CS}+c\right)\cdot \left(d\cdot \mathrm{SC}+e\right)}{{\mathrm{SR}}_{M}}$$

where SR is the sedimentation rate in 10^−2^ m/3600 s; SC is the solvent content as a percentage; and CS is the surfactant concentration in solution, g/dm^3^.

Values of the coefficients from Equation (4) for studied surfactants presented in Table 5.

The reliability of the obtained mathematical models was estimated by calculating the coefficients of nonlinear multiple correlation (3). The minimum coefficient obtained of nonlinear multiple correlation among the proposed mathematical models was 0.994.

### 3.3. Investigation of Surfactant Adsorption on Pigment

**Figure 6:**
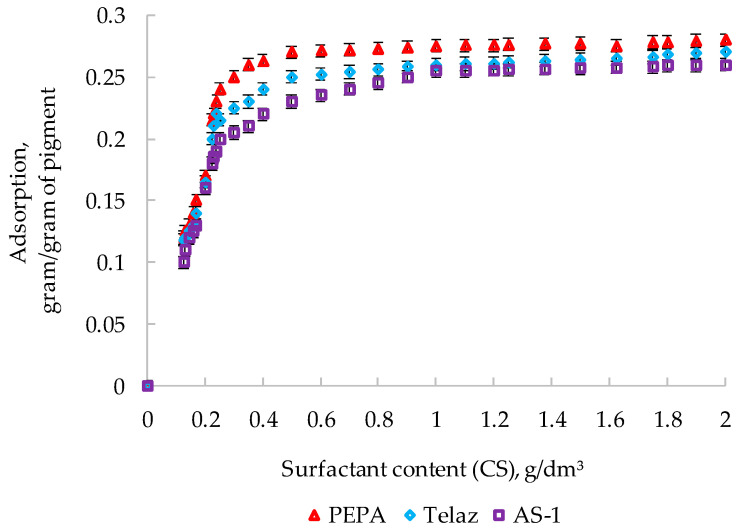
Adsorptions of surfactants in the presence of PPhS (SC10%) on the surface of aluminum pigment (at t = (20 ± 1) °C).

The effect of the surfactant type on adsorption in solutions of PPhS on aluminum pigment (at SC 10%) is shown in Figure 6.

In order to evaluate the mechanism of surfactant adsorption, the energy characteristics of the adsorption were calculated. The values of the activation energy (E_act_.) of the surfactant adsorption processes are shown in Table 6.

### 3.4. The Effects of Surfactants on the Quality of Coatings

**Figure 7:**
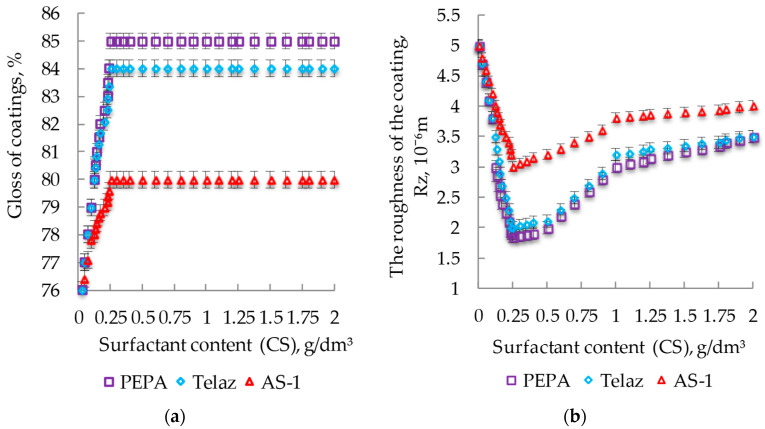
Effects of different surfactants on gloss (**a**) and roughness of coatings (**b**) at SC10%.

The effects of different surfactants on gloss are shown in Figure 7a, on their effects on the roughnesses of coatings are shown in Figure 7b.

**Figure 8:**
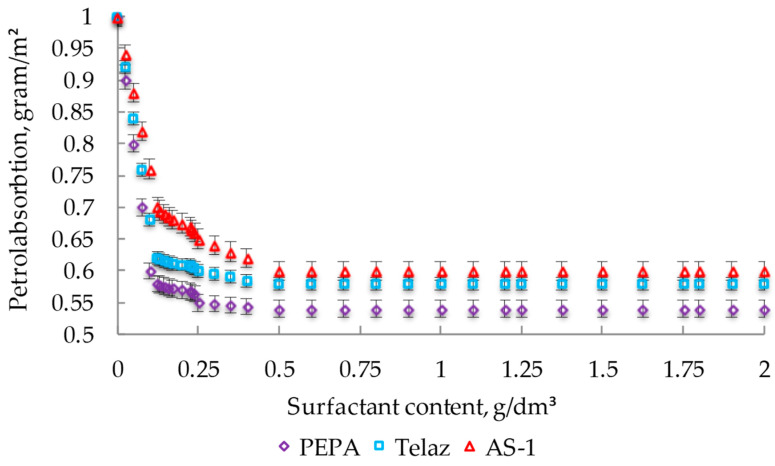
Effects of different surfactants on the petrol absorption of coatings (at SC 10%) (at t = (25 ± 5) °C).

The effects of different surfactants on the petrol absorption of coatings are shown in Figure 8.

**Figure 9:**
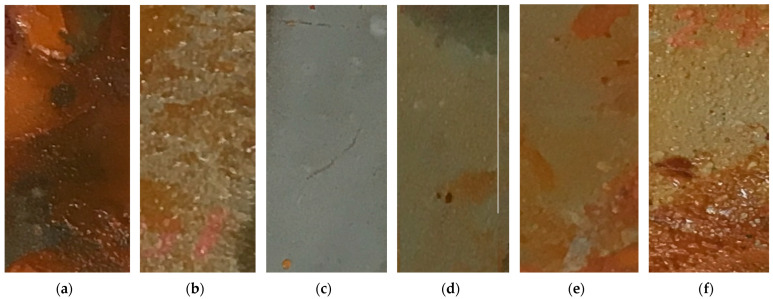
Influence of the content of PEPA additive on water resistance of the coating (held in the Caspian Sea water at 18 °C for 168 h) (Content of PEPA additive: (**a**) 0%; (**b**) 0.125%; (**c**) 0.25%; (**d**) 0.5%; (**e**) 0.875%; (**f**) 1%).

Taking into account the use of the studied silicone enamel for painting gas and oil pipelines, as well as wellhead equipment of oil wells and shut-off valves that are in contact with water of the Caspian Sea, the effect of surfactants on the resistance of the coatings of the studied enamel to the water of the Caspian Sea was studied, as shown in Figure 9.

## 4. Discussion

The introduction of surfactants into the enamel reduces the sedimentation rates of suspensions (Figure 3). This indicates an increase in the aggregative stabilities of the suspensions “aluminum pigment-toluene” (Figure 3a) and “aluminum pigment-PPhS-toluene” (Figure 3b–d). The three surfactants studied by the degree of influence on the sedimentation rate of suspensions “PPhS-toluene-aluminum pigment”/“toluene-aluminum pigment” can be arranged in a row (in descending order of stability): PEPA > Telaz > AS-1 (Figure 3). According to the results, the most effective surfactant stabilizer of suspensions in the sedimentation study was PEPA. In a pure solvent (a system without PPhS), the SR was stabilized by all three surfactants at their content level of 1 g/dm^3^; with a further increase in CS (over 1 g/dm^3^), the SR does not increase. With an increase in CS above 0.375 g/dm^3^, the SR increases, and for different surfactants, this increase in sedimentation rate is different.

With an increase in the amount of solvent (SC) in the composition, the SR naturally increases. Previously, we conducted a study of the effects of these various surfactants on the dispersion of pigment in silicone enamel [34]. It proved that all three studied surfactants (PEPA, Telaz, AS-1) significantly improved the dispersion of pigment in silicone enamel [34].

**Figure 10:**
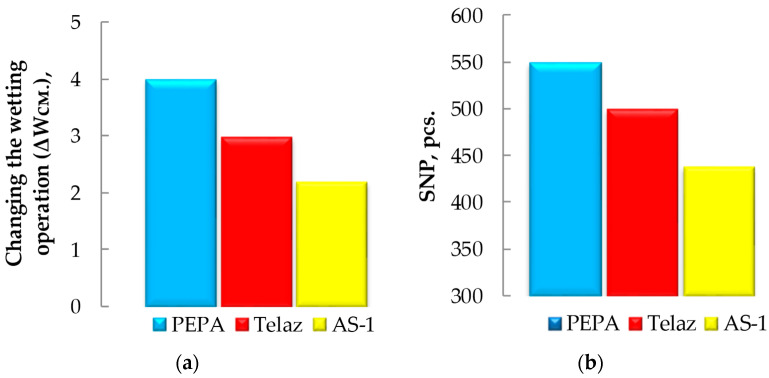
The effect of additives on the change in the wetting operation (ΔW_cm_) with respect to (**a**) the aluminum substrate (SC 10%) and (**b**) the dispersion of the pigment (SNP, pcs.), at CS 0.25 g/dm^3^ and at a temperature of 25 °C [28].

An interesting correlation was observed between SR (Figure 3), the specific number of pigment particles (Figure 10b) and the change in the wetting operation (ΔW_cm_), previously studied [28] (Figure 10a).

Among the three surfactants studied, the most effective additive in silicone enamel is PEPA, because of its maximum wetting, dispersion and stabilization effects (Figure 3 and Figure 10).

Of the three surfactants studied, the minimum values of the specific adsorption of PPhS are characteristic of PEPA; therefore, it is PEPA that is most fully adsorbed on the active centers of the aluminum pigment. The calculated values of the activation energies of all of the surfactants and PPhS in the studied temperature range indicated the chemisorption nature of adsorption on the surface of aluminum pigment (since the E_act._ is more than 8 kJ/mol) Table 6.

The introduction of surfactants into the composition caused an increase in the gloss of the coatings, with the greatest in the case of PEPA (Figure 7a). With CS PEPA at 0.25 g/dm^3^, the gloss of the coatings increased by 13% (from 75 to 85%). The introduction of surfactants into the enamel reduced the roughness of the coatings, as shown in Figure 7b. When testing with PEPA CS 0.25 g/dm^3^, the roughness of the films decreased by 2.5 times (from 5 to 2 microns). The introduction of surfactants reduced the petrol absorption of coatings by about 1.5 times (from 1 to 0.6 g/m^2^), as shown in Figure 8. The introduction of PEPA at CS 0.25 g/dm^3^ increased the resistance of coatings in the water of the Caspian Sea, thereby increasing protective characteristics significantly (Figure 9). Therefore, it can be concluded that the maximum decorative and protective characteristics of coatings are observed when the surfactant content in the enamel is 0.25 g/dm^3^.

**Figure 11:**
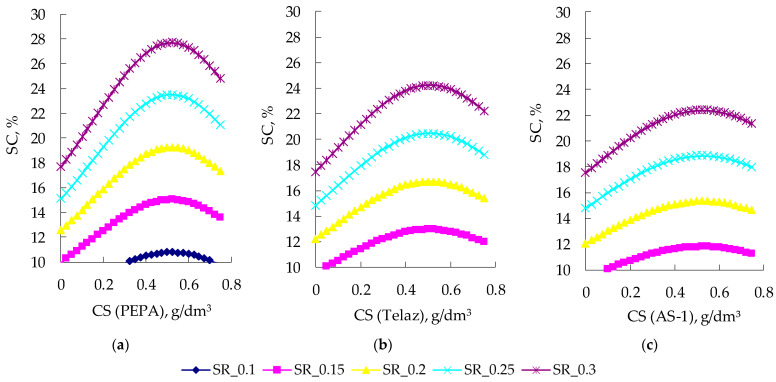
Two-factor nomograms: (**a**) SC = *f* (SR, CS (PEPA)); (**b**) SC = *f* (SR, CS (Telaz)); (**c**) SC = *f* (SR, CS (AS-1)).

Using the Taguchi method in addition to Equation (4) calculations, the optimal composition of silicone enamel (surfactant—0.375 g/dm^3^ and solvent 10%) was determined that provided maximum aggregative stability of suspensions as well as protective and decorative characteristics of coatings. For the surfactants studied, Figure 11 presents nomograms for SC (in %) graphical determination using known SR (in 10^−2^ m/3600 s) and CS (in g/dm^3^) values.

In view of the use of silicone coatings for heat resistance, the logical continuation of this study is to investigate the effect of surfactants on the supramolecular structure (domains) of coatings under repeated heating. Currently, this issue has seldom been investigated in the literature.

Currently, one of the most effective ways to reduce environmental pollution by chemical waste is to recycle the waste into useful products. In particular, oil refining waste under the trademark KON-92 that is produced in large quantities, is a valuable chemical raw material, which is currently being used mainly for heating houses in winter. We propose using this material to obtain an original additive AS-1 (Figure 1), which can be used as a dispersant and stabilizing additive for silicone enamels. The effectiveness of the AS-1 as an additive that exhibits dispersion, stabilization and wetting activities in various paint and varnish materials (pentaphthalic, silicone, urethane-alkyd, bitumen, oil, perchlorovinyl), has been confirmed by this study, and by previous research [28,29,34].

## 5. Conclusions

(1) All of the studied surfactants were found to increase the aggregative stability of suspensions, due to the joint chemisorption of resin molecules (polyphenylsyloxane) and surfactants on the pigment. The maximum aggregative stability of suspensions was achieved using PEPA, when its content in enamel was 0.25–0.375 g/dm^3^. As a result, there was a decrease in roughness (2.5 times) and petrol absorption (1.5 times), and an increase in the gloss of coatings by 13%. Using the Taguchi method, the optimal formulation of silicone enamel (surfactant—0.25 g/dm^3^ and solvent 10%) was determined that provided maximum protective and decorative characteristics to the coatings.

(2) The AS-1 additive we offer can be used as an effective stabilizing additive in industrial silicone enamels. The stabilizing ability of AS-1 is close to that of industrial additives (PAPA and Telez); however, AS-1 is synthesized from oil refining waste, which is its unsurpassed advantage, both from an economic point of view (low cost) as well as an environmental point of view (reduction in environmental pollution).

## Figures and Tables

**Table 1:** Parameters and levels used for the Taguchi method.

Factors	Level 1	Level 2	Level 3
CS (g/dm^3^)	0	0.375	0.75
SC (wt.%)	10	20	30

**Table 2:** S/N ratios of SB for the SR.

Trial	CS (g/dm^3^)	SC (wt.%)	S/N Ratio for SR, (dB)	SR, 10^−2^ m/3600 s (10^−2^ m/h)
1	0	10	15.391	0.170
2	0	20	9.119	0.350
3	0	30	5.680	0.520
4	0.375	10	18.416	0.120
5	0.375	20	11.057	0.280
6	0.375	30	7.535	0.420
7	0.75	10	18.273	0.122
8	0.75	20	10.663	0.293
9	0.75	30	7.371	0.428

**Table 3:** S/N ratios of SR by parameter level (SB).

Level	S/N Ratio	
	CS (g/dm^3^)	SC (wt.%)
1	10.063	17.360
2	12.336	10.279
3	12.102	6.862
Delta	2.273	10.498
Rank	2	1

**Table 4:** ANOVA for S/N ratios (for SR).

Factor	Degrees of Freedom	Sum of Squares	Contribution of Factor, (%)
CS (g/dm^3^)	2	9.38	5.17
SC (wt.%)	2	172.02	94.83
Total	4	181.40	100

**Table 5:** Values of the coefficients from Equation (4) for various surfactants.

№	Surfactant	*a*	*b*	*c*	*d*	*e*	SR*_M_*
1	PEPA	0.5084	−0.5284	0.3467	0.0151	−0.0348	0.2677
2	Telaz	0.4030	−0.4089	0.3467	0.0158	−0.0272	0.2878
3	AS-1	0.2880	−0.3036	0.3467	0.0159	−0.0183	0.3003

**Table 6:** Energy parameters of surfactant adsorption.

Parameter	PEPA	Telaz	AS-1
E_act._, kJ/mol	18	15	16

## Data Availability

The datasets generated during and/or analyzed during the current study are available from the corresponding author upon reasonable request.
